# Signature Movements Lead to Efficient Search for Threatening Actions

**DOI:** 10.1371/journal.pone.0037085

**Published:** 2012-05-23

**Authors:** Jeroen J. A. van Boxtel, Hongjing Lu

**Affiliations:** 1 Department of Psychology, University of California Los Angeles, Los Angeles, California, United States of America; 2 Department of Statistics, University of California Los Angeles, Los Angeles, California, United States of America; CNRS - Université Claude Bernard Lyon 1, France

## Abstract

The ability to find and evade fighting persons in a crowd is potentially life-saving. To investigate how the visual system processes threatening actions, we employed a visual search paradigm with threatening boxer targets among emotionally-neutral walker distractors, and vice versa. We found that a boxer popped out for both intact and scrambled actions, whereas walkers did not. A reverse correlation analysis revealed that observers' responses clustered around the time of the “punch", a signature movement of boxing actions, but not around specific movements of the walker. These findings support the existence of a detector for signature movements in action perception. This detector helps in rapidly detecting aggressive behavior in a crowd, potentially through an expedited (sub)cortical threat-detection mechanism.

## Introduction

The ultimate goal of perception and cognition is to enable effective interactions with the external environment. For survival purposes, humans seem specifically attuned to angry, aversive or threatening stimuli [1], [2], and appear to possess specialized neural hardware that supports these sensitivities [3], [4]. The special status of threat-related stimuli has been demonstrated using visual search paradigms. Targets that represent a *physical* threat (e.g., snakes and spiders) are rapidly detected among neutral distractors [4], [5], independent of the number of distractors, a phenomenon termed *pop-out*. The evidence for a pop-out effect for *social* threat is, however, equivocal. It has been reported that angry faces pop out among neutral faces [6], [7], suggesting that socially-threatening angry faces receive processing priority over neutral faces. However, this effect was later shown to depend on low-level stimulus differences that did not relate to threat [8], [9].

**Figure 1:**
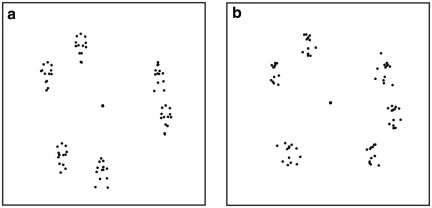
Example stimuli. (**a**) An intact boxer among walkers. (**b**) A scrambled boxer among scrambled walkers. Examples show a static frame in a target-present condition with set size 6. Each trial contained 3, 6, or 9 items. Among all items there was at most 1 target. The observers indicated whether or not the target action was present.

Rather than focusing on static images, the present study aims to examine how threat-related information affects visual search in a complex *dynamic* environment. Motion is a very powerful cue when interpreting social, emotional and communicative interactions. The usefulness of motion cues is exemplified by its efficiency in aiding identification of predators and their prey [10]. When moving dots are arranged to depict human joint movements (biological motion point-light animations, or PLAs; see Figure 1), the human visual system can derive a variety of actions (e.g., boxing, dancing, jumping jacks), and actor traits (e.g., gender) from these simplified point-light stimuli [11]–[21]. Importantly, the visual system can also derive socially important affective [22]–[24] and communicative [16], [20] information from motion cues.

It is clear that the detection of threatening actions is essential for survival and for the interpretation of social interactions, and that people seem more attuned to angry biological motion sequences than to other emotions [24]. However, it remains unknown how the human visual system manages to identify such actions in complex dynamic scenes, such as a crowd. The present paper aims to address two basic questions. First, do threatening actions receive prioritized processing among emotionally-neutral stimuli? If so, what critical information does the visual system utilize to enable such prioritized processing?

To answer these key questions, we examined an important phenomenon indicative of processing priority: pop-out for threat-related stimuli. The pop-out effect can be experimentally characterized by a measure of *search efficiency*, quantified in terms of dependence of reaction time (RT) on the number of displayed items (set size). Absence of dependence (a slope of 0 ms/item) indicates pop-out. Pop-out of a target implies that its detection may occur without the time-consuming allocation of attention. This in turn suggests that the target possesses some critical feature(s), and that perhaps specialized detection mechanisms enable the pop-out to occur [25]–[29].

Visual search paradigms have been used in biological motion perception, specifically with walking PLAs [30]–[32]. However, pop-out was not obtained between upright walkers and inverted walkers [32]. In the present paper we will focus on a comparison between threatening boxing stimuli and emotionally-neutral walker stimuli. By comparing actions that pop-out to those that do not, we can identify those critical features that enable rapid visual search [26], potentially revealing the make-up of the visual system's threat-detection mechanism.

## Methods

### Subjects

Observers were UCLA undergraduate students (age 21.5±0.5 years in Experiment 1, 20±1 years in Experiment 2). In order to prevent speed-accuracy tradeoffs, we used data from observers who reached at least 80% correct responses in the easiest condition (set size 3). We analyzed 14 (of originally 17) observers in Experiment 1 and 13 (of originally 14) observers in Experiment 2. Observers had no experience with performing boxing as a sport before. Participants received course credit for their participation and all were naïve to the purpose of the experiment. The University of California (ULCA) Institutional review board (IRB) specifically approved this study. We obtained verbal informed consent from all participants involved in our study, no records were kept as approved and required by the UCLA IRB. The study has been conducted according to the principles expressed in the Declaration of Helsinki.

### Stimuli

Action stimuli were generated from a free online motion-capture database (http://mocap.cs.cmu.edu), which provides the three-dimensional coordinates for all joints of the actors over time. We used orthogonal projections to display 13 joints in the format of point-light animations (PLAs) (the two feet, knees, hips, wrists, elbows, shoulder joints, and the head). The stimuli were displayed at 75 Hz, and screen resolution was 1024×768 pixels. A black fixation mark (size: 0.4°) was drawn at the center of the screen. The PLA could appear at any of 9 positions on an invisible circle, equally spaced around the fixation mark at a distance of 6.7°. Observers used a chin-rest to maintain a distance of 57 cm from the screen.

The point-light actors were scaled down to be 3.5° in height. Joints were displayed as black dots (diameter 0.25°) on a white background. The individual actions rotated in depth at the speed of 150°/sec. The rotation was added in order to increase the complexity of dot movements and to break the periodicity of the 2D movements in a walking sequence. This manipulation enhanced the heterogeneity of actions within each category. In addition, depth rotation helps to assess whether the experimental findings were viewpoint-independent. Even with depth rotation, all actions were still easily recognized in isolation. In pilot data we found that with non-rotating actions the same is obtained. This may not be surprising, because with the non-rotating actions, low-level confounds that may help pop-out (i.e. maximum speed, and spatial extent) are correlated with the time of the punch, and will therefore only make the pop-out stronger. Each item started at a random frame within the movie sequence and with a random viewpoint. We used actions randomly selected from two different categories: boxing (6 different movies), and walking (14 different movies).

The visual search paradigm required the short movie sequences to be loopable. In order to allow for looping, we used in-house software that performed an exhaustive search for the minimum of squared distances between matching joints between two frames. We chose the frames with the smallest mean squared distance as start and end frames, with the restriction that the action was still recognizable and the individual dots remained moving in the same direction. Mean squared distances between the selected start and end frames were not significantly different between walking and boxing movies (two-tailed t-test, p>0.05). In order to assure that the looping did not function as a cue to the subject, we performed the reverse correlation technique (see below) with the 1^st^ frame as an event frame, and no significant correlations were found. This result confirms that the looping was not influencing our data.

### Procedure

Observers searched for a boxer target among walker distractors and vice versa. Observers signaled the absence or presence of a target action by pressing one of two buttons. A beep sounded after correct responses. Each trial started with a 1-sec pause during which the completed number of trials, and total number of trials, were displayed. Observers were asked to focus on the fixation point during this period. Once the PLAs were displayed, observers were allowed to move their gaze around.

There were 2 target-distractor combinations (a boxer among walkers, or a walker among boxers), with 3 different set sizes (3, 6, or 9 items in the display), and two “presence" conditions (absent or present). Trial order was random within a block, and each block continued until each condition was correctly reported 10 times for both target-present and target-absent trials. We choose this procedure to ensure that the same number of trials (i.e. the correct ones) were used in the analysis. This procedure could potentially lead to different numbers of exposures to different conditions, when some conditions cause more errors. However, we found that the actual difference in number of trials per condition is very small. Over all 17 subjects, it ranged from, on average, 43.6 trials in 3 item conditions, to 45.2 in 6 item conditions, to 46.5 trials in 9 item conditions. If we count only to trials in which the target was present (the trials in which the subject could have learned something from increased exposure) the average number of trials ranges from 21.3 in 3 item, 21.3 in 6 item, and 21.7 trials in 9 item conditions, with all but 2 subjects having only 0 or 1 trials difference between 3-item and 9-item conditions. We therefore conclude that a difference in exposure is unable to explain our data.

Experiment 1 included two blocks in sequence, the first in which target and distractor actions were intact, and the second in which the PLAs were scrambled. Scrambling was achieved by randomly relocating each joint's *x* and *y* starting positions within a small area approximately the size of the original PLA. The scrambling was the same for all displayed items, keeping the general layout similar among all PLAs, just as it was in the intact conditions. The final scrambled figure does not look like a human action, but is still perceived as a 3D “organically-moving" volume of dots (i.e. the volume of dots gives a certain feeling of animacy [33]). Any large 3D movements (e.g. punch) in the intact actions, would also lead to large 3D movements in the scrambled actions (but because of the rotation, not necessarily corresponding to large projected 2D movements). Importantly, the scrambled version does not contain the configural motion and form information that is present in the intact actions.

Each block was divided into two subblocks, one for a walking target among boxers, and one for a boxing target among walkers. The observers started each subblock by viewing 10 sample stimuli of targets and distractors (showing side by side one sample labeled as target and one labeled as non-target, until the observer pressed a button to see the next example). Observers were instructed to closely inspect the sample stimuli. They were told that neither target items nor distractor items were all identical, but that all targets were drawn from the same category, as were all distractor stimuli. No mention was made that the samples were of human actions. Twelve practice trials followed, including 4 trials for each set size. The order of the subblocks was counterbalanced over observers.

### Data analyses

Response times in correctly identified target-present trials were used for data analyses. The search slopes and intercepts were fitted using a linear regression with response times as dependent measures. For each individual, response times for each set size were analyzed using geometric means.

## Results

Observers searched for a boxer target among walker distractors and vice versa, as shown in Figure 1.

**Figure 2:**
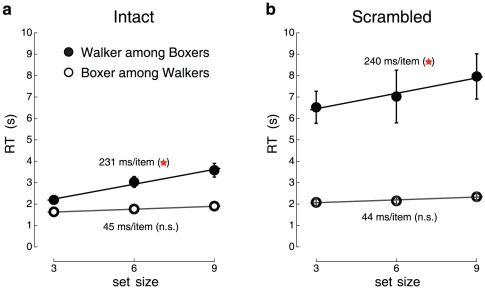
Visual search asymmetry and pop-out in intact and scrambled displays. (**a**) Reaction times (RTs) in target-present trials as a function of set size for intact point-light displays with walker targets among boxer distractors and vice versa. (**b**) RTs for scrambled point-light displays. Data points are mean ± s.e.m.

We found that boxing stimuli were rapidly detected among walker stimuli, but not the reverse (Figure 2A, B). The average slope in the intact condition for the boxing targets was not significantly different from 0 (45 ms/item, two-tailed tests: t(13) = 1.72, p>0.1; Cohen's d = 0.46). The average slope for the walking target was 231 ms/item (t(13) = 6.41, p<0.0005, Cohen's d = 1.71). Similar results were obtained for the scrambled condition. The average slope for the scrambled boxing targets (Figure 2B) was not different from 0 (slope = 44 ms/item, t(13) = 1.49, p>0.15, Cohen's d = 0.40). The average slope for the scrambled walking target was 240 ms/item (t(13) = 2.88, p<0.02, Cohen's d = 0.77). There was a significant difference in search slopes (186 ms/item, one-tailed paired t-tests: t(13) = 4.39, *p*<0.0005, Cohen's d = 1.17).

We thus found the critical result: a boxer popped out among walkers in both the intact and scrambled conditions, indicating that boxing actions do not require the focus of attention in order to be detected. Equally importantly, pop-out was specific to the boxing actions, because walkers did not pop-out among boxers. This finding suggests the existence of critical features in boxing actions that facilitate visual search [26]. Furthermore, because the pop-out was found in both intact and scrambled conditions, configural information appears not to play an essential role in rapid detection of threatening actions such as boxers. Instead, the critical features appear to be related to the movements of individual joints.

In a second experiment, we observed nearly identical results with intact and inverted conditions. However, in the inverted condition, the boxer did not pop out, even though it was still reported very rapidly (slope: 74 ms/item (t(12) = 8.77, p<0.0001, Cohen's d = 2.43). The walker slope was 253 ms/item (t(12) = 4.88, p<0.0005, Cohen's d = 1.35). These data are consistent with the first experiment, but the removal of the pop-out effect using inverted displays suggests that the critical movement feature allowing efficient search by the visual system relies on the orientation of the trajectories of joint movements. A similar dependence on orientation has previously been reported for discriminating walking directions, which is also decreased by inversion [34].

**Figure 3:**
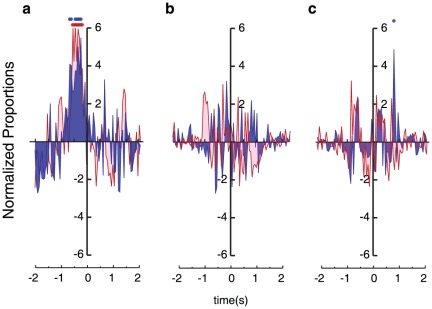
Reverse correlation results. The frequency of annotated signature movements relative to the time of a button press for intact and scrambled conditions. Punching motions (**a**) show significant correlations before the button press. Leg crossings (**b**), and leg extensions (**c**) did not correlate with button presses at any point in time before the button press. The proportions have been normalized by dividing them by the standard deviation over all proportions over time of the respective condition. Because the mean is zero, these normalized proportions are in effect Z-values. Significant correlations, corrected for false discovery rate, are indicated with filled circles (red for scrambled, blue for intact).

In order to investigate whether there are any correlations between the observers' responses and the time that signature movements occurred in action stimuli (e.g., leg-crossing and leg-extension in walking movies [13], [17], [35], and punching in boxing movies), we employed reverse-correlation analyses (i.e. response-triggered averaging: see Supporting Information S1). Figure 3 shows the results of the reverse correlation analysis relative to punching, leg crossing, and leg extensions for intact and scrambled conditions, considering 2 seconds before and after the button press. Only in the case of identifying boxing actions were there any significant correlations between observer response time and stimulus display time of presenting the signature movement. Figure 3A indicates that an observer reported the presence of boxing action 160–600 ms after viewing a punch frame. Importantly, this finding was obtained for both intact and scrambled conditions. When we bootstrapped the mean and standard deviation of a normal distribution fitted to the punching events in Figure 3, we found that the peak was closer to zero for intact (−359 ms) than in the scrambled (−448 ms) conditions (bootstrapped *p* = 0.013). In contrast to punching movements, there were no significant correlations between observers' response times and two critical walking postures when searching for walkers (i.e., leg-crossing and leg-extension; see Figure 3B and C). These results show that the visual system can readily detect a punch as a critical movement primitive. In contrast, such movement primitives were not revealed for the detection of the emotionally-neutral walking action.

One might hypothesize that boxing movements have a higher speed or motion energy than walking movements. However, because our stimuli rotated in depth, punching movements were not regularly associated with high 2D (projected) speeds. Indeed, we found that the punch detector is not simply based on a burst of motion energy associated with boxing action; a reverse correlation analysis similar to the one above (see Supporting Information S1, Figure S1) found no significant correlation between observer responses and the stimulus frame yielding the maximum inter-frame velocity when searching for boxers. There were also no significant correlations with mean speed, and maximum root mean square spatial extent.

**Figure 4:**
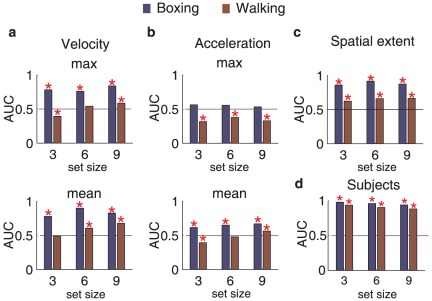
Results of the ROC analysis. Accuracy measured by the area under the ROC curve (AUC) for maximum and mean velocity (A), and acceleration (B) signals, spatial extent (C) and the observers' average performance (D). Neither velocity, acceleration or spatial extent ROC analyses reached levels comparable to human performance. No analysis yields a search asymmetry effect (an increase in difference between boxing and walking target as set size increases). These analyses show that local motion signals cannot explain our findings. Red stars indicate significant difference from chance level performance (p<.05).

Finally, we performed an ROC analysis (see Supporting Information S1) in which the model determined the optimal performance based solely on low-level motion statistic information, such as the mean and maximum speed/acceleration per displayed item in each trial. The item that was most deviant from the other was identified as the target if the deviation passed a threshold (which was varied to compute the ROC curve). If no item passed the threshold, the trial was considered a target-absent trial. We found that, based solely on 2D velocity or acceleration signals, an ideal observer is unable to perform the current task at the level that the subjects did (Figure 4). This analysis suggests that motion energy was not a confounding factor in our experiments.

One could also argue that differences in 3D motion (e.g. 3D speed and acceleration) between walkers and boxers could cause the pop-out effect. However, the 3D motion in upright and inverted actors is very similar (save for a wholesale inversion of the trajectories), and the upright boxer shows a pop-out effect while the inverted boxer does not. Therefore, it is unlikely that 3D motion per se yields the pop-out effect.

## Discussion

We have shown that the visual system performs an efficient search for threatening action stimuli. Threatening boxing actions are rapidly found, and pop out, among emotionally neutral walkers. Conversely, neutral walkers are difficult to find among threatening boxers. These findings indicate that the human visual system may be tuned to detect aggressive behavior in an otherwise non-violent crowd. But what are the critical features that facilitate the search for boxing actions, but not for walking actions?

We found that certain movements in boxer stimuli, but not walker stimuli, yielded significant correlations with observers' responses. Specifically, punch movements proceeded the time of the observer's response (Figure 3). Moreover, using the punch detector derived from the stimulus movies, it proved possible to predict human responses in searching for other boxing actions (see Supporting Information S1, Figure S2). The ability of the model to predict human responses indicates that the punch detector may function as the signature movement filter that allows for rapid detection of a boxer.

It is likely that a specialized mechanism enables the rapid detection of boxing stimuli by identifying signature movements: a punch detector. This suggestion is supported by the finding that (1) strong correlations exist between the punching movement and observers' response times, (2) pop-out depends on the orientation of actors, (3) pop-out does not dependent on local 2D and 3D motion, nor on configural information thus indicating that potential low-level confounds are not at the origin of our effects. This “punch detector" is different from the previously hypothesized “life detector" [34], in that the punch detector is viewpoint independent and not based on information from the feet, but instead based on information from the arms, because they produce the punch. We conjecture that the detectors for signature movements are likely specialized in processing motion trajectories of certain joints (in our case arm trajectories in 3D) to facilitate action perception, independent of low-level motion signals. We cannot currently pin down which arm joint is most important, but the wrist is likely to be the strongest conveyer of the punch information, because it makes the largest 3D excursion through space.

The hypothesized specificity to punching movements (and not to high-velocity ballistic motions in general) is consistent with work in neurophysiology that has shown very narrow specificities of neurons to certain arm movements in, e.g., the superior temporal sulcus (STS) [36], and premotor areas [37]. We showed here that the identified signature movements are specific to boxing actions (Figure 4), and our previous work [35] has shown that other non-threatening actions involving fast movements and ballisitic changes in velocity (e.g., running and dancing), do not yield efficient search among walking, nor do they show a search asymmetry. Therefore, the information that enables rapid search and pop-out is not dependent on fast actor movements per se. The rapid search thus appears specific to boxing actions, and possibly extents to other threatening actions. The threat-related information is likely contained in the dynamic content of the stimuli [38] but it is not simply 2D speed or spatial layout.

Accordingly, we suggest that a signature movement detector subserves rapid detection of boxing actions, which may be mediated by an expedited (sub)cortical processing route that is sensitive to threatening stimuli [1], [2], [39]. Such a mechanism has many advantages. It minimizes demand on computation resources, as it does not require a complete analysis of the entire action sequence. By enabling rapid detection of potential threatening situations, the mechanism allows the visual system to allocate greater computation resources to limited locations. This scheme of prompt detection followed by detailed action analysis makes it possible to produce adaptive behavioral responses, such as flight or adjustments in social behavior, within a brief period of time.

## Supporting Information

Supporting Information S1 Reverse Correlation, Response-triggered averaging, shows that low-level cues are not significantly correlated with the time of the button press. Additionally, ROC-analyses show that low-level motion cues cannot support an ideal observer to do the task as well as the participants did. Finally, a model that uses the time of the punch, and “punch kernels" is able to predict the time of the button press.
(DOC)

Figure S1 **Reverse correlation on maximum, average speed and maximum RMS spatial extent.** There exists no significant correlation between the time of the button press and the (a) maximum 2D speed (b) average speed, (c) RMS spatial extent. This shows that the correlation with the punch movement (Figure 3 in main document) is not due to other potential confounding factors, such as maximum or average speed (or motion energy), or spatial layout.
(EPS)

Figure S2 **Prediction data** (**a**) Peri-stimulus time histogram used as a kernel (with bin size ∼13 ms; here shown with 80-ms bin size for illustrational purposes). (**b**) Prediction of the model (line) and actual response distribution (gray area) for one boxing movie. The peak in responses around 24 s is at the time the actor ducks away for a punch from the invisible opponent (i.e., an implied punch). This peak was practically absent in responses for the inverted and scrambled conditions.
(EPS)
