# Supplementary material for: Signature Movements Lead to Efficient Search for Threatening Actions
Source: PLoS One. 2012 May 23;7(5):e37085. doi: 10.1371/journal.pone.0037085 (PMC3359369; doi:10.1371/journal.pone.0037085)
Supplement: Supporting Information S1 — Reverse Correlation, Response-triggered averaging, shows that low-level cues are not significantly correlated with the time of the button press. Additionally, ROC-analyses show that low-level motion cues cannot support an ideal observer to do the task as well as the participants did. Finally, a model that uses the time of the punch, and “punch kernels" is able to predict the time of the button press. (DOC) [file pone.0037085.s001.doc]

## Supplemental Material

### *Reverse Correlation – Response-triggered averaging*

We manually annotated each boxing movie for the frames with punches, and annotated walking movies for leg crossings, and for positions with the legs were most extended (seen from a sagittal view). Our annotations assigned a binary label (0 or 1) to each frame to indicate whether it contained a punch (frame where arm was most extended) or non-punch frame, or critical walking postures (i.e., leg-crossings and leg-extensions). The observer’s response time on each trial was used as the reference time point for each trial. We then averaged the frame labels across all trials in which the observer correctly identified the target (i.e., response-triggered averaging). The averaged binary labels over trials indicate the probability of the occurrence of a critical event at a time relative to observers’ responses. Spurious occurrence probabilities (due to different movie lengths) were removed by subtracting baseline probability (calculated by using a 5-frame moving average over simulated random responses). This analysis made it possible to assess trial-by-trial correlations between action stimulus frames and the times of observers’ responses. Significance was determined by calculating a Z-score, with the accompanying p-value at a level of 0.05.

#### *Reverse correlation on maximum, average speed and maximum RMS spatial extent*

The reverse correlation on the maximum speed was identical to the above reverse correlation. The only differences were that the frame with the maximum 2D speed was annotated as 1, while other frames were annotated as 0, and the baseline was assumed to be zero at all timepoints. We found no correlation between the maximum speed and the subjects’ responses (Figure S1)

Similarly, we performed a reverse correlation on the interframe speed (each frame was annotated with the average speed of all 13 joints), and the time of the button press, as well as a reverse correlation on the maximum root mean squared (RMS) spatial extent and a button press. We calculated the RMS spread for each frame as follows: for each dot we took the distance from it’s mean position and squared that value. The squared values for all dots in the frame were then averaged, and the square root was taken of this average. For each trial, the maximum over all frames was then taken for the ROC analysis. There were no significant correlations between observer responses and stimulus measures.

Figure S1 about here.

### *ROC analyses: low-level information does not underlie the search asymmetry*

The results of Experiments 1 and 2 revealed that configural information is not the source of the observed search asymmetry. Might low-level motion information cause the asymmetry in action search? Some previous ROC analyses have shown that point-light displays contain a great deal of information that influences certain discrimination tasks [1,2]. In order to determine the contribution of low-level motion signals, we conducted an ROC analysis on the mean and maximum speed and acceleration for Experiment 2, in which each condition had the same number of trials for correctly detecting the target actions.

We conducted a non-parametric ROC analysis on the mean and maximum 2D speed and acceleration over all joints per item. These measures were calculated along the horizontal and vertical direction independently for each displayed item. We then calculated per trial each item’s distance from the average (calculated over the remaining items in that trial), and recorded the maximum deviation. Trials in which the maximum deviation passed a threshold were marked as “target present” trials; other trials were marked as target absent. These decisions were compared to the actual presence of a target in trials, yielding hits, misses, false alarms, and correct rejections. ROC curves were constructed by varying the threshold. The area under the curve (AUC) was calculated using the standard trapezoidal numerical integration method (trapz function of MatLab).

We found no evidence for a strong involvement of low-level motion information in our observers’ search performance. First, in no instance did the ROC analysis reach the levels of search performance attained by human observers in the intact condition. Overall, velocity signals yielded the best performance, but performance generally hovered around 0.75 for boxers, and 0.5 (change level) for walkers, as measured by the area under the ROC curve (AUC). This level is significantly below the average human performance in the most difficult condition (walker among boxers, set size 9, with an AUC of about 0.87). We also analyzed the root mean squared spread of the points, and this measure showed reasonably high AUCs for boxers (about 0.87), but low performance for walkers (around 0.6). There was no clear evidence for a decrease in performance with larger set size. In fact there appeared to be an increase in performance with larger set sizes. The ROC analyses thus suggests that low-level motion signals do not underlie the visual search asymmetry. Furthermore, none of these measures correlated with the time of the press (as shown in Figure S1).

### *Prediction model*

Given the significant correlation between punching frames and observers’ responses, we developed a model to predict when people will respond during a presentation.

#### *Methods*

We devised a prediction model using peri-stimulus time histograms (PSTH). We computed PSTH using the nearest (earlier or later) annotated event to each button-press. The PSTH was smoothed with a temporal Gaussian filter (σ=4 frames) and then convolved with the annotated movie (1 for event frame, 0 otherwise) to produce a prediction. A leave-one-out procedure was employed, such that the PSTH were learned from k-1 movies (k indicates the total number of movies), and were convolved with the remaining annotated movie. This prediction procedure guarantees that the training data are independent from model prediction data. The prediction was compared to the actual responses (smoothed with Gaussian kernel, σ=15 frames). Both prediction and actual response distributions were normalized such that the area under each curve equaled 1.

#### *Results and discussion*

We used annotated punch frames as a reference to calculate peri-stimulus time histograms (PSTH) by counting the frequency of responses within a certain time interval from the annotated events (see Figure S2A for an example). When we ran the prediction model on our data, the model explains 33% (median) of the variance over all intact boxing movies (see example in Figure S2B), 33% (median) for the scrambled movies, and 32% for the inverted movies. Interestingly, when we trained the model on the intact conditions, and tested on the scrambled and inverted conditions, we found that intact conditions better explained the scrambled data than the inverted data (mean difference over movies is about 10%-points). Similarly, the PSTHs (i.e., kernels; see Methods) were more highly correlated between intact and scrambled than between intact and inverted conditions (comparing correlation coefficients: t(5)=2.21, p=0.039, one-tailed paired t-test). These findings are consistent with the experimental result that the intact and scrambled conditions are more similar is terms of pop-out than either of them compared to the inverted condition.

Figure S2 about here

## Supplemental References

1. Gold JM, Tadin D, Cook SC, Blake R (2008) The efficiency of biological motion perception. Percept Psychophys 70: 88-95.

2. Pollick FE, Kay JW, Heim K, Stringer R (2005) Gender recognition from point-light walkers. J Exp Psychol Hum Percept Perform 31: 1247-1265.
